# Effects of obesity on outcomes of laparoscopic transabdominal preperitoneal inguinal hernia repair: a retrospective analysis

**DOI:** 10.20452/wiitm.2024.17896

**Published:** 2024-08-07

**Authors:** Dawid Golik, Przemysław Sroczyński, Krzysztof Jędras, Grzegorz Dobkowski, Michał R. Janik

**Affiliations:** Military Institute of Aviation Medicine, Warszawa, Poland

**Keywords:** body mass index, inguinal hernia, obesity, recurrence rate, transabdominal preperitoneal patch plasty

## Abstract

**INTRODUCTION:**

Inguinal hernia repair, particularly the transabdominal preperitoneal (TAPP) technique, is common worldwide. Obesity (body mass index [BMI] ≥30 kg/m^2^) can influence surgical outcomes, potentially resulting in longer operative time, higher complication rate, and prolonged hospital stay.

**AIM:**

This study aimed to evaluate the impact of obesity on surgical outcomes following laparoscopic TAPP inguinal hernia repair.

**MATERIALS AND METHODS:**

We retrospectively reviewed data from patients who underwent laparoscopic TAPP inguinal hernia repair between September 2021 and December 2023. We included patients aged 18 years or older who had elective unilateral TAPP repair for primary inguinal hernia. The patients were categorized based their BMI as obese (BMI ≥30 kg/m^2^) and nonobese (BMI <⁠30 kg/m^2^). Outcomes assessed included recurrence rate, surgical site infections (SSIs), operative time, and length of hospital stay.

**RESULTS:**

We analyzed 201 patients of whom 30 (14.8%) were obese and 171 (85.2%) were nonobese. Recurrence rates were 6.67% in the obese and 2.35% in the nonobese patients (*P* = 0.222). No SSIs were observed in the obese patients, as compared with 1.76% in the nonobese individuals (*P* = 1). Mean (SD) operative time was 78.87 (31.88) minutes for the obese and 70.28 (27.25) minutes for the nonobese patients (*P* = 0.203). Mean (SD) hospital stay was 3.13 (0.35) days for the patients with and 3.05 (0.28) days for those without obesity (*P* = 0.086).

**CONCLUSIONS:**

There were no significant differences in recurrence rates, SSIs, operative time, or hospital stay between the obese and nonobese patients. Appropriate surgical expertise and perioperative management can result in comparable outcomes for both groups. Further research is recommended to understand the impact of obesity on hernia recurrence.

## INTRODUCTION

Inguinal hernia repair is one of the most often performed surgical procedures worldwide, with the transabdominal preperitoneal (TAPP) technique being a widely accepted minimally invasive approach.^1^ Despite its popularity, various patient-specific factors, such as obesity, can significantly influence surgical outcomes. Obesity, defined by body mass index (BMI) of 30 kg/m^2^ or higher, is a growing global health concern that has been associated with increased surgical risks, including longer operative time, higher complication rate, and prolonged hospital stay.^2^^; ^^3^^; ^^4^^; ^^5^ The relationship between obesity and surgical outcomes in inguinal hernia repair remains complex and multifaceted. Previous studies have highlighted technical challenges posed by increased amounts of adipose tissue, such as reduced visibility and accessibility during laparoscopic procedures, which can potentially compromise the efficiency and effectiveness of the TAPP technique.^6^^; ^^7^ Additionally, obesity is often associated with comorbid conditions, such as diabetes and cardiovascular diseases, which may further complicate postoperative recovery.^8^ Given the rising prevalence of obesity and its potential implications on surgical practice, it is crucial to understand how this condition specifically affects the outcomes of TAPP inguinal hernia repair.

## AIM

This study aimed to evaluate the impact of obesity on various surgical outcomes following laparoscopic TAPP inguinal hernia repair. Specifically, we assessed the recurrence rate of inguinal hernia, hypothesizing that the obese patients (BMI ≥30 kg/m^2^) have a higher recurrence rate than the nonobese ones (BMI <⁠30 kg/m^2^). We also examined the incidence of surgical site infections (SSIs), with the expectation that the obese patients shall experience a higher rate of SSIs. Additionally, we compared the operative time, hypothesizing that it is longer in the obese patients, and determined the length of hospital stay, with the assumption the obese individuals have a longer hospital stay than the nonobese ones.

## MATERIALS AND METHODS

### Study design

This study was designed as a retrospective observational analysis set in our surgical center. We reviewed clinical data of patients who underwent laparoscopic TAPP inguinal hernia repair from September 2021 to December 2023. The study followed STROBE guidelines for observational studies.^9^

### Participants

The study included patients who were at least 18 years old and underwent elective unilateral TAPP repair for primary inguinal hernia. The exclusion criteria included 1) bilateral inguinal hernia repair, 2) open inguinal hernia repair, 3) emergency hernia repair, and 4) insufficient follow-up data.

The participants were selected from the hospital’s electronic medical records. Follow-up was conducted through routine postoperative visits and review of medical records up to 1 year postsurgery. The patients were divided into 2 groups based on their BMI, that is, the study group with BMI equal to or above 30 kg/m² and the control group with BMI below 30 kg/m². No additional matching criteria were applied, as this was not a matched study.

## Procedure description

Laparoscopic TAPP inguinal hernia repair was performed under general anesthesia. Pneumoperitoneum was established, and 3 trocars were inserted in a standard configuration. The peritoneum was incised to access the preperitoneal space, where the hernia sac was identified and reduced. A synthetic mesh, with a minimum size of 15 cm × 10 cm, was placed to cover the hernia defect, ensuring it extended beyond the defect margins by at least 5 cm. For hernias classified as M3 or L3 according to the European Hernia Society (EHS) classification, the mesh was secured with glue or sutures.^10^^; ^^11^^; ^^12^ The peritoneum was then closed over the mesh to restore the anatomy. Operative time, postoperative complications, and patient recovery were meticulously recorded.

### Outcomes

The primary outcomes assessed in this study included recurrence rate, defined as the incidence of hernia recurrence within 1 year postsurgery; SSIs, defined as infections at the surgical site within 180 days postsurgery; operative time, measured in minutes from the initial incision to the completion of the surgery; and length of hospital stay, measured as the number of days from surgery to discharge.

### Data sources and measurement

Data were sourced from electronic medical records. BMI was calculated using standard height and weight measurements. Operative time, length of hospital stay, and recurrence rates were directly extracted from surgical and follow-up records. SSIs were diagnosed based on clinical criteria and documented in postoperative notes.

### Bias

Efforts to address potential sources of bias included standardized data collection procedures and consistent follow-up protocols for all patients.

### Study size

The study size was determined by the number of eligible patients meeting the inclusion criteria within the specified time frame. Sample size calculations were not performed due to the retrospective design of the study.

### Statistical analysis

Quantitative variables (operative time, length of hospital stay) were handled using means and SD. Grouping was based on BMI categories to compare the obese and nonobese patients.

Data analysis was conducted using SAS Studio statistical software (SAS Institute Inc., Cary, North Carolina, United States). Descriptive statistics, such as means, SD, and percentages, were calculated for demographic and procedure-related variables. Categorical variables were compared between the 2 groups using the χ^2^ test or the Fisher exact test. The Wilcoxon signed-rank test was employed to compare continuous variables. Statistical significance was determined at *P* value below 0.05.

### Ethical considerations

This retrospective study utilized existing medical data that had been fully anonymized, ensuring that no patient could be identified. As a result, approval from the institutional ethics committee was not required. Patient confidentiality and data privacy were strictly maintained throughout the study. Informed consent was waived due to the retrospective nature of the analysis.

## RESULTS

### Baseline characteristics

We analyzed a total of 201 patients who underwent inguinal TAPP repair. Their baseline characteristics are provided in TABLE 1. As many as 30 individuals (14.8%) were classified as obese, while the remaining 171 patients (85.2%) served as controls. The mean (SD) age of the participants was 54.03 (14.82) years. Mean (SD) body weight of the patients in the study group was 101.3 (10.63) kg, mean height was 175.93 (13.23) cm, and mean BMI reached 32.96 (4.35) kg/m². Approximately 90% of the study group patients were male.

**TABLE 1:** Baseline characteristics of the patients

Variable	Study group (n = 30)	Control group (n = 171)
Age^a^, y	54.03 (14.82)	59.497 (14.72)
Body weight, kg	101.27 (10.63)	78.379 (11.08)
Height^a^, cm	175.93 (13.23)	175.488 (8.3)
BMI^a^, kg/m^2^	32.96 (4.35)	25.367 (2.46)
Men, %	90	88.89

Data are presented as mean (SD) unless indicated otherwise. ; **a ***P *<⁠0.05 ; Abbreviations: BMI, body mass index

Respective mean (SD) parameters in the control group were 59.5 (14.72) years, 78.38 (11.08) kg, 175.49 (8.29) cm, and 25.37 (2.46) kg/m². As many as 88.89% of the control group participants were men.

Additionally, right-sided hernia was confirmed in 53.22% of the patients with obesity and 76.67% of the controls. Differences in age and hernia site were significant. TABLE 2 presents hernia types according to the EHS classification.

**TABLE 2:** European Hernia Society inguinal hernia classification^9^

Classification	General	Study group	Control group
Medial (M)			
Overall	36.58	25	37.8
1	6.67	–	7.14
2	26.67	–	26.67
3	66.67	100	64.29
Lateral (L)			
Overall	58.54	75	56.76
1	20.83	33.3	16.67
2	68.5	66.7	54.17
3	16.67	–	16.67
Femoral (F)			
Overall	4.87	–	5.46
1	100	–	100
2	–	–	–
3	–	–	–

Data are shown as percentage of patients.

### Surgical outcomes

The recurrence rate within 1 year postsurgery was 6.67% in the study group and 2.35% in the control group (*P* = 0.22). No SSIs were observed in the study group, whereas in the control group their rate was 1.76% (*P* = 1). The mean (SD) operative time was 78.87 (31.88) minutes and 70.28 (27.25) minutes in the study and control group, respectively (*P* = 0.2). The mean (SD) length of hospital stay was 3.13 (0.35) days in the study group and 3.05 (0.28) days in the controls (*P* = 0.086). The findings for the patients with and without obesity are summarized in TABLE 3.

**TABLE 3:** Surgical outcomes

Variable	Study group (n = 30)	Control group (n = 171)	*P* value
Operative time, min	78.87 (31.88)	70.28 (27.25)	0.2
Length of hospital stay, d	3.13 (0.35)	3.05 (0.28)	0.09
Surgical site infection, %	0	1.76	>0.99
Recurrence rate, %	6.67	2.35	0.22

Data are shown as mean (SD) or percentage of patients.

In summary, while the obese patients showed a higher recurrence rate, longer operative time, and longer hospital stay, the differences were not significant when compared to the nonobese group. The difference observed for SSI rate was also insignificant.

The post hoc logistic regression analysis indicated that obesity (odds ratio [OR], 3.082; 95% CI, 0.495–19.205; *P* = 0.228), age (OR, 0.978; 95% CI, 0.927–1.032; *P* = 0.413), or hernia side (left vs right; OR, 1.745; 95% CI, 0.315–9.668; *P* = 0.524) were not significant predictors of hernia recurrence following TAPP repair. The C-statistic of 0.682 indicates moderate predictive power of the model. While the OR for obesity suggested a potential increase in the risk of recurrence, wide confidence intervals and high *P* values reflected a lack of significance. Future research in larger groups and additional covariates may help better understand the relationship between these factors and hernia recurrence.

## DISCUSSION

This study aimed to assess the impact of obesity on surgical outcomes in patients undergoing laparoscopic TAPP for inguinal hernia repair. Prior studies suggest that the incidence of inguinal hernia is lower among obese patients, potentially due to a protective effect of increased abdominal fat.^13^^; ^^14^ However, obesity may pose challenges during surgical procedures, potentially leading to higher complication rate and extended hospital stay.^15^^; ^^16^^; ^^17^ Inguinal hernia surgery can significantly improve patient physical functioning and emotional well-being. Specifically, patients often experience enhanced physical capabilities and better emotional functioning, while their perceptions of general health and energy levels are generally satisfactory.^18^ This suggests that hernia surgery should be offered to all patients with a clinically detectable hernia, regardless of BMI. However, in individuals with very high BMI, a multimodal approach that includes both bariatric surgery and hernia repair should be considered. For patients with severe obesity and ventral hernias suitable for laparoscopic repair, this combined strategy has proven to be safe and to yield good short-term outcomes. Incorporating bariatric surgery can address the underlying obesity, potentially lowering the risk of hernia recurrence and enhancing overall surgical success.^16^

The outcomes assessed in our study were recurrence rate, SSIs, operative time, and length of hospital stay. Our findings provide valuable insights into the influence of obesity on these critical surgical outcomes.

The recurrence rate was higher in the obese than nonobese patients (6.67% vs 2.35%), although the difference was not significant. This result is consistent with a previous study suggesting that obesity may contribute to higher recurrence rates due to increased intra-abdominal pressure and technical challenges during surgery.^17^ The lack of statistical significance in our study may be attributed to the sample size, and further research in larger cohorts may be necessary to clarify this relationship.

No SSIs were observed in the study group, whereas in the control group the SSI rate reached 1.76%. This finding contrasts with a general expectation that obesity is associated with a higher risk of SSIs due to factors such as impaired wound healing and increased skin folds.^19^ The discrepancy may be due to effective perioperative care and stringent infection control measures at our institution. Nonetheless, it underscores the importance of meticulous surgical technique and postoperative care in minimizing infection risks.

The mean operative time was longer for the obese patients (78.87 vs 70.28 min), but the difference was insignificant. This aligns with the existing literature indicating that obesity can increase operative time due to technical difficulties, such as reduced visibility and maneuverability during laparoscopic procedures. The lack of a significant difference in our study suggests that with experienced surgeons and optimized surgical techniques, the impact of obesity on operative time can be mitigated.

The hospital stay was slightly longer for the obese than nonobese patients (3.13 vs 3.05 days), but again the result did not reach statistical significance. This finding is in line with previous research^20^ indicating that obesity may prolong hospital stay due to more complex postoperative care needs. However, the marginal difference observed in our study highlights the potential for enhanced recovery protocols to standardize care irrespective of BMI.

Our study contributes to the ongoing debate regarding the impact of obesity on surgical outcomes in TAPP inguinal hernia repair. While some studies have demonstrated significant adverse effects of obesity, our findings suggest that with appropriate surgical expertise and perioperative management, these effects can be minimized. The lack of significant differences in recurrence rates, SSIs, operative time, and length of hospital stay between the obese and nonobese patients highlights the potential for tailored surgical strategies to address the challenges posed by obesity. Our results correspond with a previous study investigating the effect of obesity on the safety of cholecystectomy, which demonstrated that obesity was not a risk factor in terms of surgical safety.^21^

### Limitations

Our study has several limitations. The retrospective design may introduce selection bias and limit the ability to control all potential confounding variables. The relatively small sample size, particularly in the study group, may reduce the power to detect significant differences. Additionally, the single-center setting may limit the generalizability of the findings.

## CONCLUSIONS

In conclusion, while obesity presents certain challenges for TAPP inguinal hernia repair, our study suggests that with skilled surgical practice and rigorous perioperative care, outcomes in obese patients can be comparable to those in nonobese individuals. Future research with larger, multicenter cohorts is warranted to further elucidate the impact of obesity on surgical outcomes and to develop optimized management protocols for this patient population.
